# Bisdemethoxycurcumin alleviates LPS-induced acute lung injury via activating AMPKα pathway

**DOI:** 10.1186/s40360-023-00698-3

**Published:** 2023-11-20

**Authors:** Huifang Li, Qi Zou, Xueming Wang

**Affiliations:** 1 Department of respiration medicine, Huangzhou District People’s Hospital, Huanggang, 438000 Hubei China; 2 Department of intensive care unit, Huangzhou District People’s Hospital, Zhonghuan Road 31, Huanggang, 438000 Hubei China

**Keywords:** Bisdemethoxycurcumin, Acute lung injury, AMPKα, Oxidative stress, Inflammation

## Abstract

**Objective:**

Inflammation and oxidative stress contribute to the pathogenesis of acute lung injury (ALI), and subsequently result in rapid deterioration in health. Considering the indispensable role of bisdemethoxycurcumin (BDMC) in inflammation and oxidative stress, the present study aims to examine the effect of BDMC on sepsis-related ALI.

**Methods:**

C57BL/6 mice were administered with BDMC (100 mg/kg) or an equal volume of vehicle, and then injected with lipopolysaccharides (LPS) to induce ALI. We assessed the parameters of lung injury, inflammatory response and oxidative stress in lung tissues. Consistently, the macrophages with or without BDMC treatment were exposed to LPS to verify the effect of BDMC in vitro.

**Results:**

BDMC suppressed LPS-induced lung injury, inflammation and oxidative stress in vivo and in vitro. Mechanistically, BDMC increased the phosphorylation of AMPKα in response to LPS stimulation, and AMPK inhibition with Compound C almost completely blunted the protective effect of BDMC in LPS-treated mice and macrophages. Moreover, we demonstrated that BDMC activated AMPKα via the cAMP/Epac pathway.

**Conclusion:**

Our study identifies the protective effect of BDMC against LPS-induced ALI, and the underlying mechanism may be related to the activation of cAMP/Epac/AMPKα signaling pathway.

**Supplementary Information:**

The online version contains supplementary material available at 10.1186/s40360-023-00698-3.

## Introduction

Acute lung injury (ALI) is a serious respiratory distress with high morbidity and mortality in clinical patients [1–3]. Sepsis is a primary pathogenic factor of ALI, and sepsis-related ALI is characterized as vascular permeability, pulmonary edema and intractable hypoxemia. Despite a wide variety of risk factors for sepsis-induced ALI, pioneering studies have revealed that the progression of ALI is generally associated with uncontrolled inflammatory response and excessive generation of reactive oxygen species (ROS) [4]. Moreover, elevated inflammatory cytokines and ROS were associated with increased mortality in patients with septic ALI [5]. Accordingly, the inhibition of inflammation and oxidative stress represents a typical therapeutic strategy for sepsis-induced ALI.

Adenosine 5’-monophosphate-activated protein kinase alpha (AMPKα) is a pivotal energy-sensed kinase, and plays critical roles in metabolic disorder, mitochondrial biology, inflammation, and oxidative stress [6, 7]. Several conditions favor that the depletion of energy levels, such as hypoxia, inhibits the activity of AMPKα. Unexpected, the activity of mammalian AMPKα is remarkably suppressed in LPS-induced ALI characterized as pulmonary hypoxia [8, 9]. In contrast, activating AMPKα confers significant benefits to sepsis-induced ALI. Liu et al. observed that buformin mitigated LPS-induced ALI via activating AMPKα in vivo [10]. Further findings from Lv et al. revealed that AMPKα activation dramatically reduced oxidative stress and inflammation in ALI mice [11]. NFE2 like bZIP transcription factor 2 (NRF2) is a central transcriptional factor that plays critical roles in preserving redox balance through facilitating the expression of anti-oxidative genes. Meanwhile, the activation of AMPKα/NRF2 pathway dramatically suppressed LPS-induced inflammation, oxidative stress and ALI [10, 12, 13]. Cyclic adenosine 3′,5′-monophosphate (cAMP) is a ubiquitous and essential intracellular second messenger involved in a wide range of physiological and pathological processes [14]. A consensus view for the interaction between cAMP and AMPKα has been reached [15]. Currently, two classical effectors of cAMP have been identified to mediate AMPKα activation, including protein kinase A (PKA) and exchange protein directly activated by cAMP (Epac). Epac belongs to the family of guanine-nucleotide exchange factors for the Ras-like GTPases, including Rap1 and Rap2, and plays critical roles in activating AMPKα (26,941,424). Fu et al. found that Epac activation by cAMP facilitated the activation of Rap1/MEK pathway, and subsequently elevated the phosphorylation of AMPKα through LKB1 (21,220,320). Meanwhile, Laurent et al. revealed that Calcium/Calmodulin Dependent Protein Kinase Kinase 2 (CaMKK2) was required for the activation of AMPKα by Epac (25,411,381). Interestingly, Park et al. also reported that the elevation of cAMP by resveratrol increased intracellular Ca2 + levels, and subsequently activated CaMKK2, thereby increasing the phosphorylation of AMPKα [16]. Moreover, cAMP mediated AMPKα activation has been pharmacologically exploited for the treatment of inflammation, oxidative stress and ALI [15, 17, 18]. Therefore, targeting theses molecular pathways may help to identify novel therapeutic candidates for ALI treatment.

Curcumin, a polyphenol component of turmeric, has been widely studied for its anti-inflammatory and anti-oxidative effects [19]. Recently, bisdemethoxycurcumin (BDMC), a demethoxy derivatives of curcumin, has attracted considerable attention due to its high bioavailability and excellent aqueous solubility [20]. Experimental evidences showed that BDMC suppressed LPS-induced inflammatory responses and mitigated intestinal damage in broilers [21]. Moreover, BDMC possessed mitochondrial protective properties and decreased LPS-induced oxidative stress via activating the mitochondrial anti-oxidative system [22]. In addition, BDMC was reported to activate AMPKα to sensitize non-small cell lung cancer to chemotherapeutic agents [23–25]. Although the anti-oxidative and anti-inflammatory properties of BDMC are well documented, whether BDMC administration could prevent LPS-related ALI remains unclear. With these findings in mind, we herein aim to investigate the role of BDMC in LPS-induced ALI and validate the underlying mechanisms.

## Materials and methods

### Chemical and reagents

BDMC (#B6938, purity ≥ 98.6%), LPS (#L2880) from *Escherichia coli* O55: B5, ApopTag Plus In Situ Apoptosis Fluorescein Detection Kit (#S7111) and 2ʹ5ʹ-dideoxyadenosine (2′5′-ddAdo), an inhibitor of adenylyl cyclase, were purchased from Sigma-Aldrich (St. Louis, MO, USA). Antibodies against total p65 (t-p65, #8242), phosphorylated p65 (p-p65, #3033), t-AMPKα (#5831), p-AMPKα (#2535) and GAPDH (#2118) were obtained from Cell Signaling Technology (Danvers, MA, USA). The anti-SOD2 (#ab68155), anti-PCNA (#ab92552) and NRF2 Transcription Factor Assay Kit (#ab207223) were purchased from Abcam (Cambridge, MA, USA). Compond C (CpC), an inhibitor of AMPKα, and PKA inhibitor (H89) were obtained from MedChemExpress (Monmouth Junction, NJ, USA). The myeloperoxidase (MPO) assay kit, malondialdehyde (MDA) assay kit, total superoxide dismutase (SOD) assay kit and glutathione (GSH) assay kit were all purchased from Nanjing Jiancheng Bioengineering Institute (Nanjing, China). Small interfering RNA against Epac and the scramble RNA (si*Epac* or si*RNA*) were synthesized by RiboBio (RiboBio Co. Ltd, Guangzhou, China).

### Animal

8-10-week-old C57BL/6 mice were purchased from the institute of Laboratory Animal Science, Chinese Academy of Medical Sciences and Peking Union Medical College (Beijing, China). All mice were bred under specific pathogen-free conditions and fed with chow and water ad libitum. To generate sepsis-induced ALI model, mice were intratracheally injected with a single dose of LPS (5 mg/kg) and maintained for 12 h. An equal volume of saline was injected as the negative control. To investigate the role of BDMC, mice were intragastrically administered with BDMC (100 mg/kg/day) for 3 days prior to LPS instillation [26]. For AMPKα inhibition, mice were intraperitoneally injected with CpC (20 mg/kg, every other day) for 3 times prior to BDMC treatment [27]. This study was approved by the Animal Research Ethics Committee of our hospital, and also adhered to the ARRIVE guidelines. All mice were sacrificed 12 h post-LPS stimulation. The animals were grouped according to a random number table. All investigators were blinded to the group allocation until the experimental endpoint.

### Lung functional determination

Lung function was determined with the Emka WBP pulmonary function test system (Connecticut, CT, USA). The airway resistance and pulmonary ventilation were collected and recorded by the detection system.

### Histological examination

To measure lung injury, paraffin-embedded lung slices were exposed to H&E staining to quantify morphologic changes, and inflammation score was calculated to evaluate lung injury as previously described [28]. Briefly, lung injury was assessed from five aspects: hemorrhage, neutrophils in the alveolar space, hyaline membranes, pertinacious debris filling the airspaces and septal thickening, and graded from 0 to 4. No damage is graded to 0, damage less than 25% is graded to 1, damage ranging from 25 to 50% is graded to 2, damage ranging from 50 to 75% is graded to 3, damage over 75% is graded to 4. The inflammation score was identified by three independent pathologist blindly. Cell apoptosis was measured using the ApopTag Plus In Situ Apoptosis Fluorescein Detection Kit according to the manufacturer’s instructions. Apoptotic index was calculated as the ratio of TUNEL + nuclei to total nuclei [29–31].

### Blood gas analysis

Arterial blood samples were collected from mouse carotid arteries. The partial pressure of carbon dioxide (PaCO_2_), sodium bicarbonate (HCO_3_^−^) and partial pressure of oxygen (PaO_2_) were measured automatically by a fully automated blood gas analyzer.

### MPO activity

Fresh lung tissues were homogenized and centrifuged to remove the insoluble material on ice. Then, the MPO activity was assessed with a commercial kit according to the instructions.

### Lung wet to dry ratio

To measure lung wet to dry ratio, fresh lung tissues were excised and weighed immediately (wet weigh), which were then placed into a 60 °C oven for 96 h to get the constant dry weight. Finally, the wet to dry ratio of lung were calculated as an index of pulmonary edema.

### Cell culture and treatment

Primary mouse peritoneal macrophages (MPMs) were isolated according to a previous study [32]. In brief, the peritoneal cavity was rinsed with phosphate buffered saline (PBS) for 3 times to collect the macrophages-enriched solution, which was then centrifuged to obtain the MPMs. Next, these cells were cultured in RPMI 160 medium with 10% fetal bovine serum (FBS). To imitate ALI model in vitro, macrophages were treated with LPS (100 ng/mL) for 6 h. To determine the role of BDMC, cells were pretreated with BDMC (10 µmol) for 12 h according to a previous study [33]. To verify the involvement of cAMP/AMPKα axis, cells were pre-incubated with 2′5′-ddAdo (200 µmol/L), H89 (10 µmol/ml) or CpC (20 µmol/L) for 12 h before BDMC incubation [34–36]. Besides, for Epac silence, cells were pre-transfected with si*Epac* (50nmol/L) or si*RNA* using a Lipo6000™ transfection reagent according to previous reports [37, 38].

### Western blot

The lung tissues and cells were collected and homogenized with RIPA lysis buffer. The protein concentrations were determined by a BCA Protein Assay Kit (Thermo Fisher, Waltham, MA) and 20 µg of total proteins were separated by SDS-PAGE and transferred to PVDF membranes (Millipore Corp, billerica, MA, USA). After 1 h of blocking, the membranes were incubated with primary antibodies at 4 °C overnight. On the next day, the membranes were incubated with HRP-conjugated secondary antibodies at room temperature for 1 h and then visualized using electrochemiluminescence reagents (Millipore, Boston, MA, USA). To save the antibody and membranes, the blots were cut prior to hybridization with antibodies according to the molecular weights provided by the antibody datasheet, and the images with clear edges and markers of molecular weights of three replicates were provided in Supplementary Information. The relative protein levels were quantified by Image J software [39, 40].

### ELISA detection

Tissue homogenates and cell medium were prepared for the measurement of interleukin-1β (IL-1β), IL-6 and tumor necrosis factor-α (TNF-α) levels using commercially available ELISA kits (R&D Systems, USA) according to the manufacturer’s instructions.

### Collection and analysis of bronchoalveolar lavage fluid (BALF)

BALF was collected as previously reported [41]. In brief, the thorax and the cervical trachea of mice was exposed after euthanasia. Then, the main trachea and left bronchus were ligated, followed by the irrigation with PBS for 3 times from right lungs. The obtained BALF was centrifuged at low temperature to collect cells, which were counted with trypan blue exclusions.

### Oxidative stress determination

Freshly prepared lung homogenates or macrophages lysates were incubated with DCFH-DA (50 µmol/L, Sigma-Aldrich, St. Louis, MO, USA) for 30 min at room temperature in the dark. After being washed for 3 times with PBS, fluorescent intensities were examined at an excitation/emission wavelength of 485/535 nm. The levels of MDA, total SOD activity, NRF2 activity and GSH in lung tissues or cells were assessed according to the manufacturer’s instructions as previously reported [42, 43].

### Biochemical analysis

The levels of cAMP and PKA activity in lung samples were detected by the commercial kits (Abcam Cambridge, UK) according to the manufacturer’s instructions.

### Statistical analysis

Data in the present study were analyzed by SPSS 23.0 software. An unpaired Student’s *t-*test was used to assess the difference between two groups, while statistical differences among three or more groups were assessed by one-way analysis of variance (ANOVA), followed by a Tukey post hoc test. All data were expressed as the mean ± standard deviation (SD). *P* < 0.05 indicates that the data are statistically different.

## Results

### BDMC administration alleviates LPS-induced ALI in mice

**Fig. 1:**
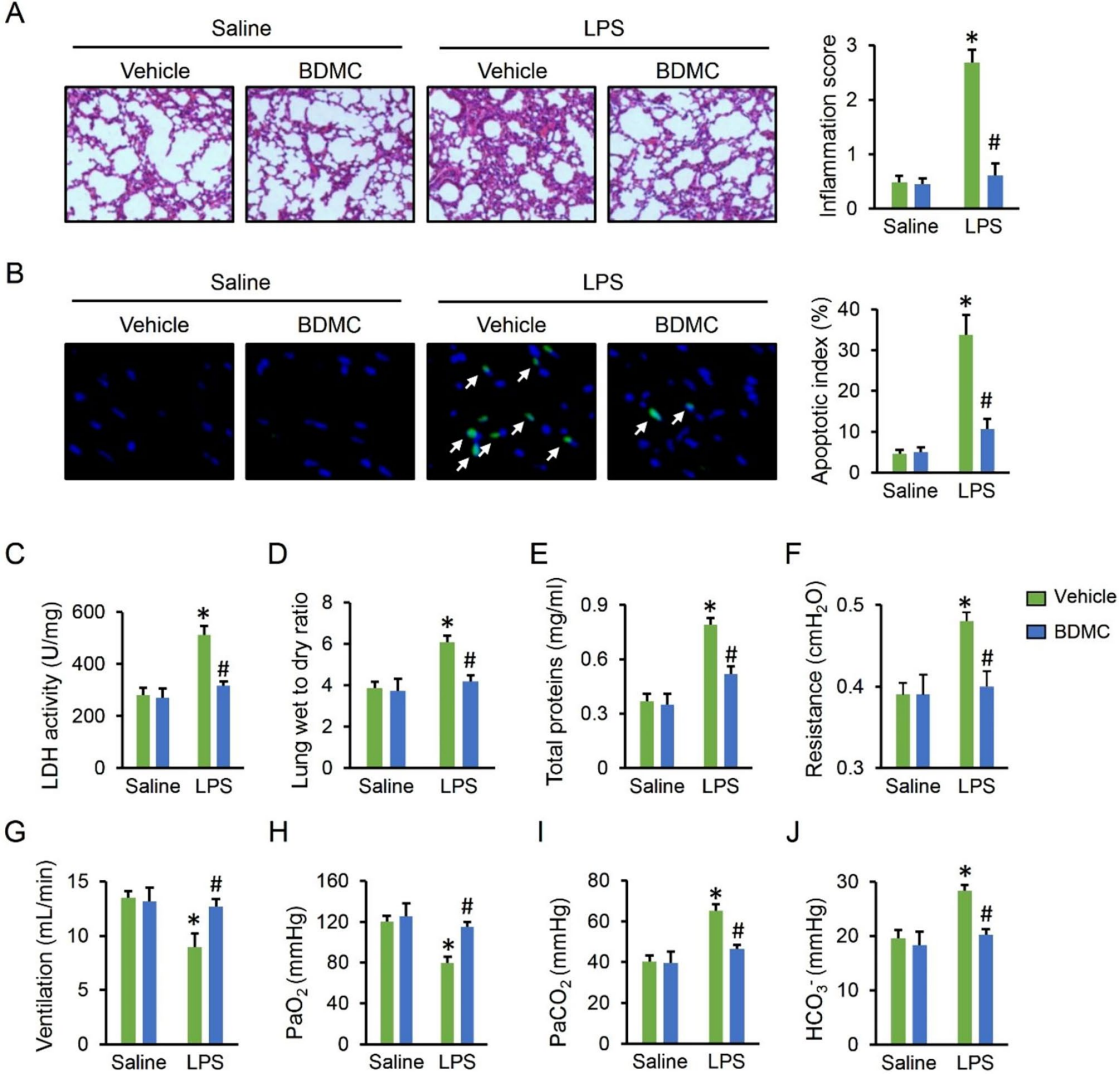
**BDMC administration alleviates LPS-induced ALI in mice**. Mice were intragastrically administered with BDMC (100 mg/kg/day) for 3 days, and then intratracheally injected with a single dose of LPS (5 mg/kg). Mice were sacrificed 12 h post-LPS injection with the lungs collected for further examination. (**A**) H&E staining and lung inflammation score were used to detect the lung histopathological changes (n = 6). (**B**) TUNEL staining and apoptotic index were used to evaluate cell apoptosis (n = 6). (**C**) The LDH activity in lungs (n = 6). (**D**) The lung wet to dry ratio (n = 6). (**E**) The total proteins in BALF (n = 6). (**F**-**G**) The pulmonary function as determined by airway resistance and pulmonary ventilation (n = 6). (**H**-**J**) The arterial blood gas analysis of PaO_2_, PaCO_2_ and HCO_3_^−^ (n = 6). Values represent the mean ± SD. *P<0.05 versus Saline + Vehicle; ^#^*P*<0.05 versus LPS + Vehicle

As shown in Fig. 1A, LPS injection resulted in pulmonary congestion, thickening of the alveolar wall and inflammatory infiltration in murine lungs, which were dramatically alleviated by BDMC administration. LPS-induced cell apoptosis was also prevented in the presence of BDMC (Fig. 1B). Accordingly, the elevated LDH activity in LPS-injured lungs was significantly decreased by BDMC (Fig. 1C). Meanwhile, BDMC administration improved LPS-induced pulmonary edema in mice, as judged by the wet to dry ratio (Fig. 1D). Also, the concentrations of total protein in BALF from ALI mice were evidently decreased by BDMC administration (Fig. 1E). In accordance with the reduced lung injury, BDMC also decreased the airway resistance and increased lung ventilation in LPS-treated mice (Fig. 1F-G). Besides, the alterations of PaO_2_, PaCO_2_ and HCO_3_^−^ further revealed the protective effects of BDMC administration against LPS-induced pulmonary dysfunction (Fig. 1H-J). Accordingly, these data indicate that BDMC administration alleviates sepsis-related ALI in mice.

### BDMC administration inhibits inflammatory responses in ALI mice

**Fig. 2:**
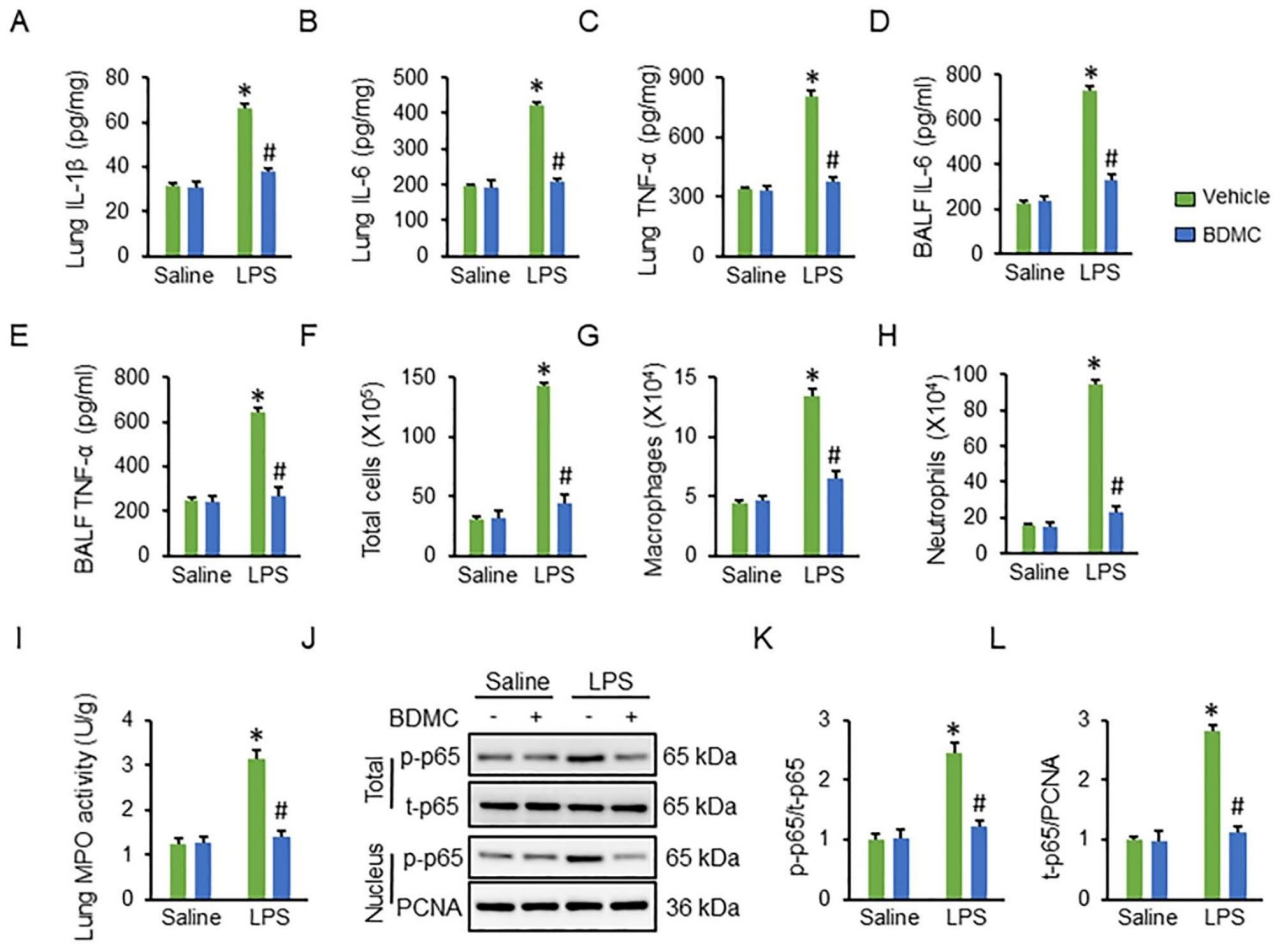
**BDMC administration inhibits inflammatory responses in ALI mice**. Mice were intragastrically administered with BDMC (100 mg/kg/day) for 3 days, and then intratracheally injected with a single dose of LPS (5 mg/kg). Mice were sacrificed 12 h post-LPS injection with the lungs collected for further examination. (**A**-**C**) The levels of inflammatory cytokines were assessed in murine lungs (n = 6). (**D**-**E**) The levels of inflammatory cytokines were assessed in BALF (n = 6). (**F**-**H**) The infiltrations of total cells, macrophages and neutrophils in BALF (n = 6). (**I**) The MPO activity in lung tissues (n = 6). (**J**-**L**) The western blot images and quantitative data of p65 phosphorylation and nuclear translocation (n = 6). Values represent the mean ± SD. *P<0.05 versus Saline + Vehicle; ^#^*P*<0.05 versus LPS + Vehicle

Considering the pivotal role of inflammation in LPS-induced ALI, we next evaluated the protective effect of BDMC on inflammation in LPS-treated mice. As shown in Fig. 2A-E, the inflammatory cytokines (IL-1β, IL-6 and TNF-α) in lung and BALF from ALI mice were significantly decreased by BDMC treatment. Moreover, the increased infiltrations of macrophages, lymphocytes and neutrophils to LPS-injured lungs were remarkably alleviated in those with BDMC protection (Fig. 2F-H). The activity of MPO, an index of neutrophils infiltration, was also inhibited by BDMC in response to LPS stimulation (Fig. 2I). NF-κB p65 acts as a key transcriptional regulator that orchestrates the expression of various inflammatory genes. As expected, LPS-elicited p65 phosphorylation and nuclear translocation were reduced with BDMC administration (Fig. 2J-L). Therefore, we conclude that BDMC administration inhibits inflammatory responses in ALI mice.

### BDMC administration alleviates oxidative stress in ALI mice

**Fig. 3:**
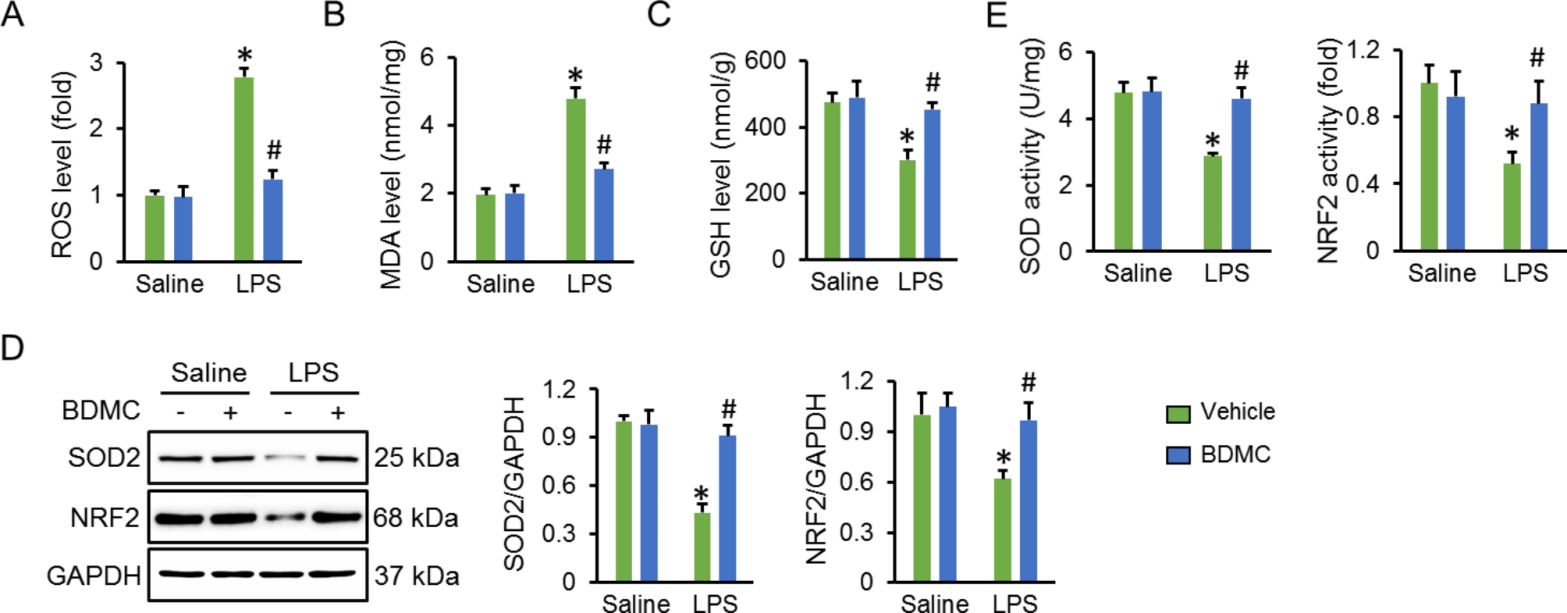
**BDMC administration alleviates oxidative stress in ALI mice**. Mice were intragastrically administered with BDMC (100 mg/kg/day) for 3 days, and then intratracheally injected with a single dose of LPS (5 mg/kg). Mice were sacrificed 12 h post-LPS injection with the lungs collected for further examination. (**A**) The ROS generation in lung tissues were assessed by a DCFH-DA probe (n = 6). (**B**-**C**) The levels MDA and GSH in murine lungs (n = 6). (**D**) The western blot images and relative statistical results of NRF2 and SOD2 (n = 6). (**E**) The levels of total SOD activity and NRF2 activity (n = 6). Values represent the mean ± SD. *P<0.05 versus Saline + Vehicle; ^#^*P*<0.05 versus LPS + Vehicle

Oxidative stress is essential for the pathogenesis of ALI. As shown in Fig. 3A, BDMC administration decreased ROS generation in LPS-treated mice. Accordingly, lipid peroxidation in LPS-injured lungs was also ameliorated by BDMC treatment, as determined by the reduced MDA levels (Fig. 3B). GSH and SOD play indispensable roles to scavenge excessive free radicals and subsequently prevent oxidative damage. Interestingly, we observed that BDMC administration preserved the levels of GSH, SOD2 protein and total SOD activity in LPS-injured lungs (Fig. 3C-E). NRF2 is a central transcriptional factor to induce the expression of anti-oxidative genes in response to oxidative stress. As shown in Fig. 3D-E, the protein level and transcriptional activity in LPS-injured lungs were elevated by BDMC administration. Taken together, we determine that BDMC administration alleviates oxidative stress in ALI mice.

### BDMC treatment inhibits LPS-induced inflammation and oxidative stress in macrophages

**Fig. 4:**
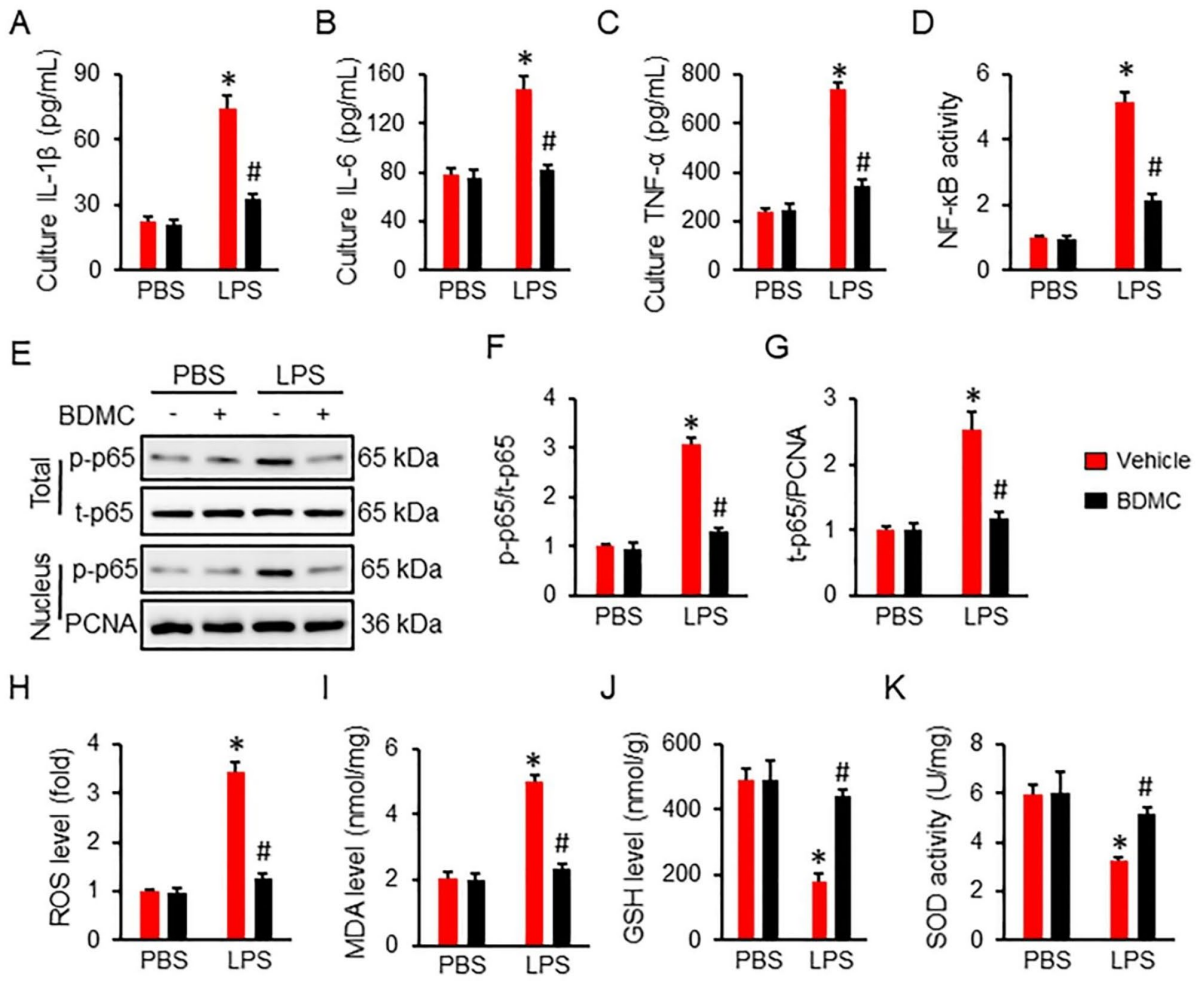
**BDMC treatment inhibits LPS-induced inflammation and oxidative stress in macrophages**. Primary mouse peritoneal macrophages were isolated and pretreated with BDMC (10 µmol) for 12 h prior to LPS insult (100 ng/mL). (**A**-**C**) The levels of inflammatory cytokines in cell medium (n = 6). (**D**) NF-κB activity in macrophages (n = 6). (**E**-**G**) The western blot images and quantitative data of p65 phosphorylation and nuclear translocation (n = 6). (**H**) The ROS generation in macrophages were assessed by a DCFH-DA probe (n = 6). (**I**-**J**) The levels MDA and GSH in macrophages (n = 6). (**K**) The levels of total SOD activity in macrophages (n = 6). Values represent the mean ± SD. *P<0.05 versus PBS + Vehicle; ^#^*P*<0.05 versus LPS + Vehicle

Macrophages mediate both innate and adaptive immune responses, and play a central role in LPS-induced ALI. As shown in Fig. 4A-C, the increased inflammatory cytokines in LPS-stimulated macrophages, including IL-1β, IL-6 and TNF-α, were suppressed in by BDMC treatment. Moreover, BDMC incubation significantly alleviated LPS-induced activation of NF-κB p65, as evidenced by the decreased activity, phosphorylation and nuclear translocation of NF-κB p655 (Fig. 4D-G). In line with the data in vivo, LPS-triggered increases of ROS and MDA were evidently mitigated in BDMC-treated macrophages (Fig. 4H-I). The decreased levels of GSH and total SOD activity in LPS-stimulated macrophages were also increased by BDMC (Fig. 4J-K). These data provide solid evidence that BDMC treatment inhibits LPS-induced inflammation and oxidative stress in macrophages.

### BDMC treatment protects against LPS-induced ALI via activating AMPKα

**Fig. 5:**
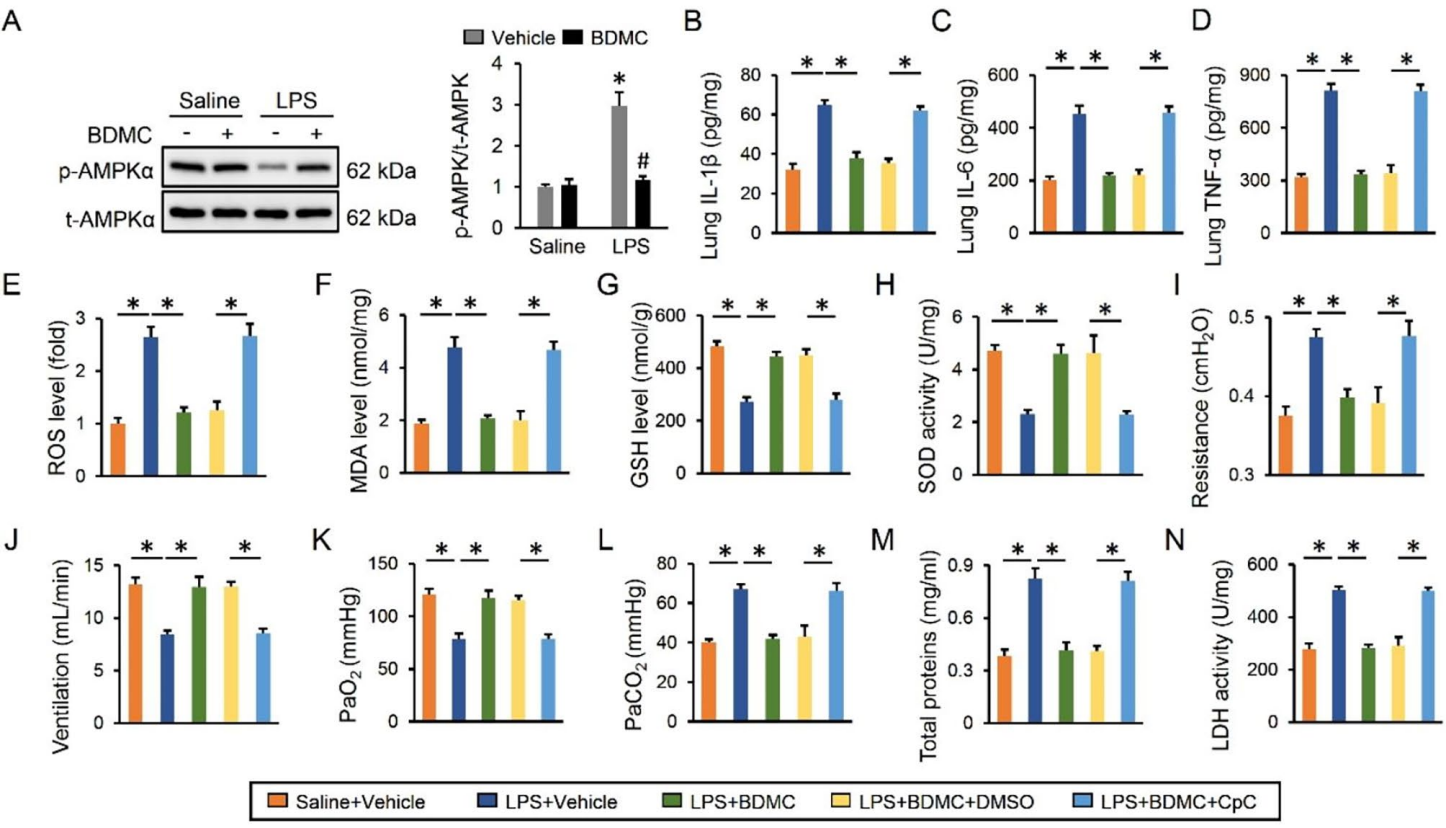
**BDMC treatment protects against LPS-induced ALI via activating AMPKα**. Mice were intragastrically administered with BDMC (100 mg/kg/day) for 3 days, and then intratracheally injected with a single dose of LPS (5 mg/kg). Mice were sacrificed 12 h post-LPS injection with the lungs collected for further examination. (**A**) The western blot images and quantitative data about AMPKα phosphorylation were presented (n = 6). For AMPKα inhibition, mice were intraperitoneally treated with CpC (20 mg/kg) once two days for 3 times before BDMC administration. (**B**-**D**) The levels of IL-1β, IL-6 and TNF-α in lung tissues (n = 6). (**E**-**H**) The levels of ROS, MDA, GSH and total SOD activity were detected in lung tissues (n = 6). (**I**-**J**) The pulmonary function as determined by airway resistance and pulmonary ventilation (n = 6). (**K**-**L**) The arterial blood gas analysis of PaO_2_ and PaCO_2_ (n = 6). (**M**) The total proteins were determined in BALF (n = 6). (**N**) The LDH activity in lung tissues (n = 6). Values represent the mean ± SD. *P < 0.05 versus the matched group. In Fig. 5A, *P < 0.05 versus Saline + Vehicle, ^#^*P* < 0.05 versus LPS + Vehicle

AMPKα is a stress-activated kinase and plays critical roles in controlling inflammation and oxidative stress. As shown in Fig. 5A and Figure S1A, we observed that the decreased AMPKα phosphorylation in LPS-stimulated mice or macrophages was increased by BDMC treatment. To elucidate the involvement of AMPKα in BDMC-mediated protective effects, we treated mice with CpC, an inhibitor of AMPKα. As expected, the anti-inflammatory effect of BDMC during LPS-induced ALI was repressed by CpC (Fig. 5B-D). Meanwhile, the reduced levels of ROS, MDA and increased levels of GSH, SOD activity in BDMC-treated ALI mice were almost abolished by CpC (Fig. 5E-H). More importantly, we found that AMPKα inhibition also negated BDMC-elicited beneficial effects against LPS-induced pulmonary dysfunction, as evidenced by the increased airway resistance, PaCO_2_ and decreased lung ventilation, PaO_2_ (Fig. 5I-L). Accordingly, the improved lung injury in BDMC-treated ALI mice was also abolished by AMPKα inhibition, as assessed by the increased levels of BALF total proteins and LDH activity (Fig. 5M-N). Together, our findings reveal that BDMC treatment protects against LPS-induced ALI via activating AMPKα.

### AMPK inhibition blunts the protective effects of BDMC in macrophages

To further confirm the role of AMPKα, macrophages were pretreated with CpC to repress AMPKα activation in vitro. Consistent with the data in vivo, we observed that the anti-inflammatory and anti-oxidative effects of BDMC in LPS-stimulated macrophages were abolished by AMPKα inhibition (Figure S1B-I). Taken together, we demonstrate that AMPK inhibition blunts the protective effects of BDMC in macrophages.

### BDMC treatment activates AMPKα via cAMP/Epac signaling pathway

**Fig. 6:**
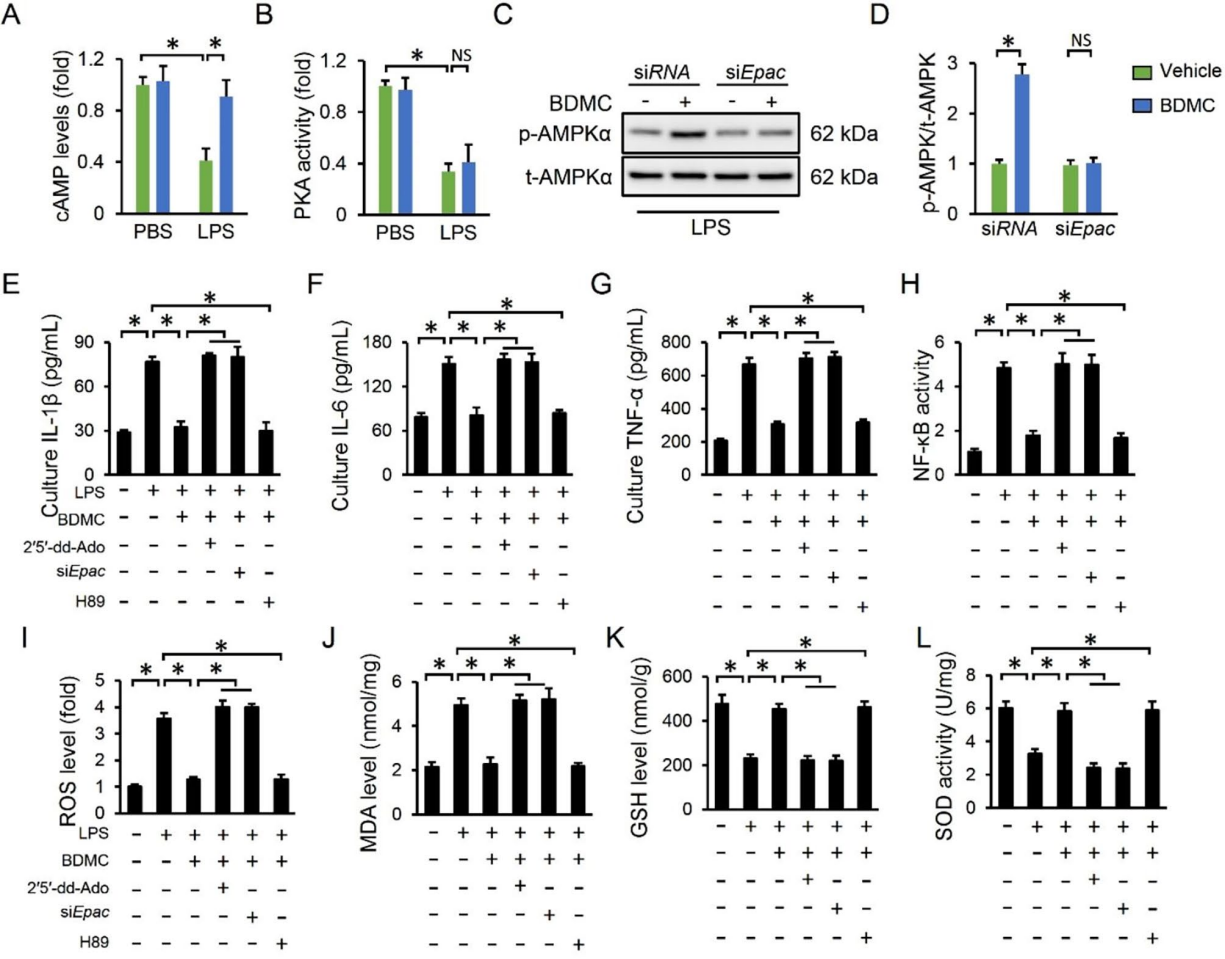
**BDMC treatment activates AMPKα via cAMP/Epac signaling pathway**. Primary mouse peritoneal macrophages were isolated and pretreated with BDMC (10 µmol) for 12 h prior to LPS insult (100 ng/mL). (**A**-**B**) The levels of cAMP and PKA activity were assessed (n = 6). To knock down Epac, cells were pre-transfected with si*Epac* (50 nmol/L) or si*RNA* using Lip0 6000™. (**C**) The AMPKα phosphorylation was determined (n = 6). Cells were pretreated with 2′5′-ddAdo (AC inhibitor, 200 µmol/L), H89 (PKA inhibitor, 10 µmol/mL) or si*Epac* before BDMC stimulation. (**E**-**G**) The levels of IL-1β, IL-6 and TNF-α in cell medium (n = 6). (**H**) The NF-κB activity in macrophages (n = 6). (**I**-**L**) The levels of ROS, MDA, GSH and total SOD activity in cells (n = 6). Values represent the mean ± SD. *P < 0.05 versus the matched group. NS means no significance

We next sought to explore the possible mechanisms mediating AMPKα activation by BDMC. Previous studies implied that cAMP-mediated AMPKα activation plays central roles in LPS-related inflammatory responses. As shown in Fig. 6A, we found that the cAMP level was decreased upon LPS stimulation, yet preserved by BDMC in macrophages. PKA and Epac are well-designed downstream factors of cAMP pathway. Intriguingly, BDMC failed to increase the PKA activity in LPS-treated cells (Fig. 6B). As shown in Fig. 6C-D, AMPKα activation by BDMC in LPS-incubated cells was blocked by si*Epac*. Therefore, we speculated that BDMC activated AMPKα via cAMP/Epac pathway, and subsequently prevented LPS-induced inflammation, oxidative stress and ALI. To further verify the involvement of cAMP/Epac pathway, cells were pretreated with 2’5’-dd-Ado, si*Epac* or H89 to inhibit cAMP, Epac or PKA, respectively. As shown in Fig. 6E-L, BDMC-mediated anti-inflammatory and anti-oxidative effects were almost completely abolished by 2’5’-dd-Ado or si*Epac*, but not H89. Together, we conclude that BDMC activates AMPKα via cAMP/Epac signaling axis in LPS-induced ALI.

## Discussion

ALI is a life-threatening illness with a high morbidity and mortality in critically ill patients [44]. A variety of pharmacological agents have been demonstrated to protect against ALI, while failed in clinical trials. Therefore, effective pharmacological therapies for ALI have emerged as an urgent paradigm. Sepsis is a leading cause (6–42%) of ALI, with a higher mortality rate as compared to other causes of ALI [45, 46]. In the present study, we found that BDMC treatment prevented LPS-induced ALI and suppressed inflammation and oxidative stress in vivo and in vitro. Mechanistically, BDMC increased AMPKα phosphorylation, while AMPKα inhibition abrogated the protective effects of BDMC in vivo and in vitro. Moreover, we observed that BDMC activated AMPKα via cAMP/Epac signaling pathway in LPS-insulted macrophages. Therefore, BDMC may be a novel therapeutic reagent for the treatment of sepsis-related ALI.

The innate immune system serves as the first line of defense to protect host against pathogens infections. The variety of immune cells recognize the damage, and subsequently produce several cytokines and chemokines to regulate both the pulmonary innate and adaptive immunity. Excessive inflammation in the lung is considered to be the key factors in the pathogenesis of ALI. The uncontrolled inflammatory response led to increased infiltrations of neutrophil and macrophages, accompanied with elevated intrapulmonary and systemic releases of pro-inflammatory cytokines and chemokines, including IL-1β, IL-6 and TNF-α. Previous studies reported that the enhanced inflammatory response was associated with a poor prognosis of ALI-related mortality. Recently, Fe-Cur NP nanozymes were demonstrated to be a promising therapeutic agent for ALI treatment via alleviating inflammation and oxidative stress [47]. Herein, we found that BDMC prevented LPS-induced infiltrations of inflammatory cells to the lungs. Additionally, LPS-induced releases of inflammatory cytokines were also repressed by BDMC treatment. ALI patients exhibit elevated chemokine levels in their BALF, which further exacerbate neutrophil infiltration into the lungs [48]. Therefore, the measurement of inflammatory cells and cytokines in BALF is of great significance for ALI patients. As expected, we observed that BDMC administration decreased the levels of inflammatory cells and cytokines in BALF of LPS-treated mice. In the initial phase of ALI, the injured alveolar-capillary membrane leads to a dysregulated flux of protein-enriched fluids into the alveolar space. Indeed, ALI patients usually develop severe pulmonary edema, refractory hypoxemia and respiratory failure. In the present study, we observed that BDMC improved pulmonary edema and decreased the levels of total proteins in BALF of ALI mice. MPO is a member of the peroxidases superfamily that is mainly expressed in neutrophils and monocytes [49]. Elevated MPO levels during ALI are associated with increased inflammation and oxidative stress. Moreover, the long-term hypoxia in response to ALI also disturbs the redox balance and results in elevated ROS generation, which further increases vascular endothelial permeability and subsequently promotes pulmonary edema [50]. A recent study verified that GPA peptide prevented cercal ligation and puncture-related ALI via decreasing ROS generation, emphasizing the pathogenic role of oxidative stress in sepsis-related ALI [51]. In this study, we demonstrated that BDMC decreased the levels of MPO activity, ROS, and increased the levels of GSH as well as total SOD activity. Mitochondria are the primary source of free radicals, and previous studies have demonstrated that BDMC could target mitochondria to prevent oxidative damage [22, 30]. In our study, we found that BDMC treatment significantly increased the anti-oxidative capacity of LPS-stimulated lungs and macrophages in vivo and in vitro. Further studies are demanded to investigate the involvement of mitochondria in this process.

AMPKα is increasingly recognized as a pivotal target in restraining inflammatory response and oxidative stress in response to LPS. Kikuchi and his colleagues found that AMPKα was a protective kinase during sepsis, and that AMPKα deletion in male mice significantly aggravated sepsis-induced organ damages, including the lung tissue [52]. NLRP3 inflammasome is required for the synthesis, maturation and releases of multiple pro-inflammatory cytokines. Interestingly, Liu et al. reported that AMPKα activation repressed NLRP3 inflammasome in LPS-induced ALI [10]. NF-κB p65 is a key transcriptional factor whose activation promotes the expression of inflammatory cytokines. It was reported that AMPKα activation could repress inflammatory responses through inhibiting NF-κB p65 activation [53, 54]. In the present study, we observed that BDMC alleviated LPS-induced inflammation via activating AMPKα. Elevated ROS levels dramatically disturb mitochondrial integrity and may regulate AMPKα phosphorylation via inducing energy stress. Conversely, AMPKα activation stimulates the expression of multiple anti-oxidative genes via controlling the expression of various anti-oxidative transcriptional factors. Chen et al. discovered that intrinsic activation of AMPKα could inhibit the generation of ROS and maintain mitochondrial homeostasis during hypoxia and reoxygenation stress in cardiomyocytes [55]. Moreover, Song et al. found that geniposide prevented sepsis-induced oxidative stress via activating AMPKα in heart [56]. Likewise, we found that BDMC prevented LPS-induced oxidative stress via activating AMPKα. More importantly, AMPKα inhibition abolished the anti-inflammatory and anti-oxidative effects of BDMC both in vivo and in vitro. AMPKα has been proved to be regulated at multiple levels. Recently, the cAMP/AMPKα axis has been suggested to be involved in several pathologies, including inflammation, oxidative stress, obesity and diabetes [15]. Besides, various cAMP-elevating agents were demonstrated to protect against alveolar injury and acute respiratory distress syndrome [57]. Hamidiee et al. found that curcumin increased cAMP levels and subsequently modulated mitochondrial biogenesis through a cAMP/PKA/AMPKα-dependent manner [58]. We herein found that cAMP was responsible for BDMC-mediated AMPKα activation. More importantly, the downstream target Epac, instead of PKA, was required for AMPKα activation by BDMC.

In conclusion, we found that BDMC administration prevented LPS-induced ALI via repressing inflammation and oxidative stress. BDMC treatment activated cAMP/Epac axis and upregulated AMPKα phosphorylation to restrain LPS-induced inflammation and oxidative stress in ALI. We speculate that BDMC may be a promising therapeutic agent for LPS-induced ALI treatment.

### Electronic supplementary material

Below is the link to the electronic supplementary material.

Supplementary Material 1

## Data Availability

The data underlying this study are available on reasonable request to the corresponding author.

## Abbreviations

ALI: Acute lung injury
AMPKα: Adenosine 5’-monophosphate-activated protein kinase alpha
BDMC: Bisdemethoxycurcumin
BALF: Bronchoalveolar lavage fluid
CpC: Compond C
cAMP: Cyclic adenosine 3′,5′-monophosphate
ELISA: Enzyme-linked immunosorbent assay
EPAC: exchange protein directly activated by cAMP
IL-1β: Interleukin-1β
LPS: Lipopolysaccharides
MDA: Malondialdehyde
MPMs: Mouse primary peritoneal macrophages
ROS: Reactive oxygen species
SOD: Superoxide dismutase
