# Supplementary material for: Bisdemethoxycurcumin alleviates LPS-induced acute lung injury via activating AMPKα pathway
Source: BMC Pharmacol Toxicol. 2023 Nov 20;24:63. doi: 10.1186/s40360-023-00698-3 (PMC10662695; doi:10.1186/s40360-023-00698-3)

**Supplementary materials**

**
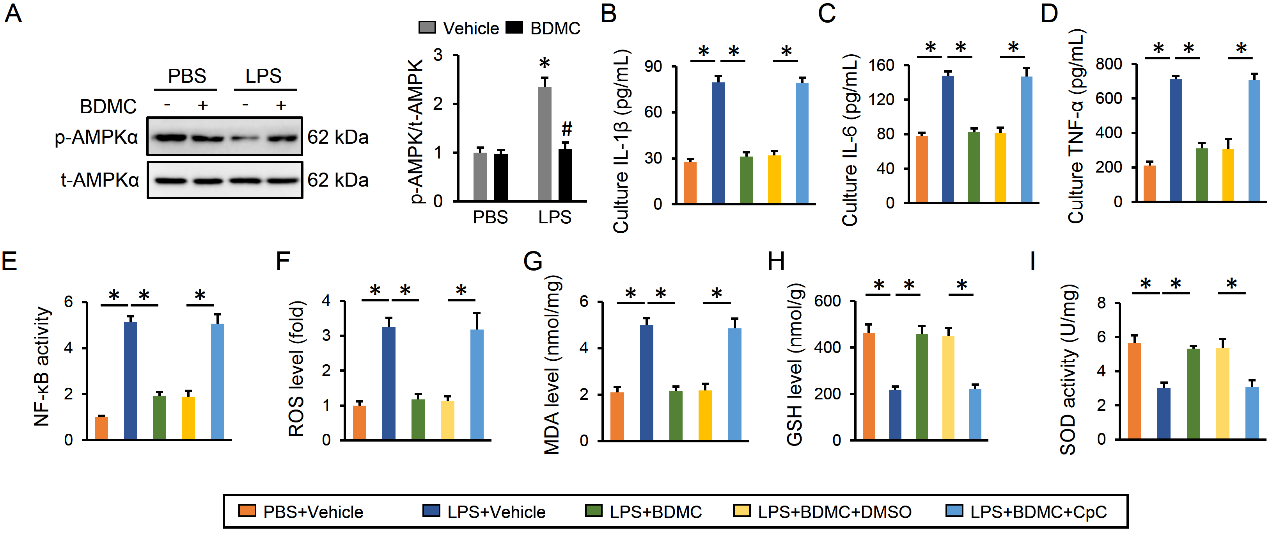
**

**Figure S1 BDMC exerts the anti-inflammatory and anti-oxidative effects via activating AMPKα in vitro.** Primary mouse peritoneal macrophages were isolated and pretreated with BDMC (10 μmol) for 12 h prior to LPS insult (100 ng/mL). (A) The levels of AMPKα phosphorylation were assessed (n=6). For AMPKα inhibition, cells were pretreated with CpC (20 μmol/L) for 12 h before BDMC stimulation. (B-D)The levels of IL-1β, IL-6 and TNF-α in cell medium (n=6). (E) The NF-κB activity in macrophages (n=6). (F-I) The levels of ROS, MDA, GSH and total SOD activity in cells (n=6). Values represent the mean ± S D. **P* < 0.05 versus the matched group. In Fig S1A, **P* < 0.05 versus PBS + Vehicle, ^#^*P* < 0.05 versus LPS + Vehicle.


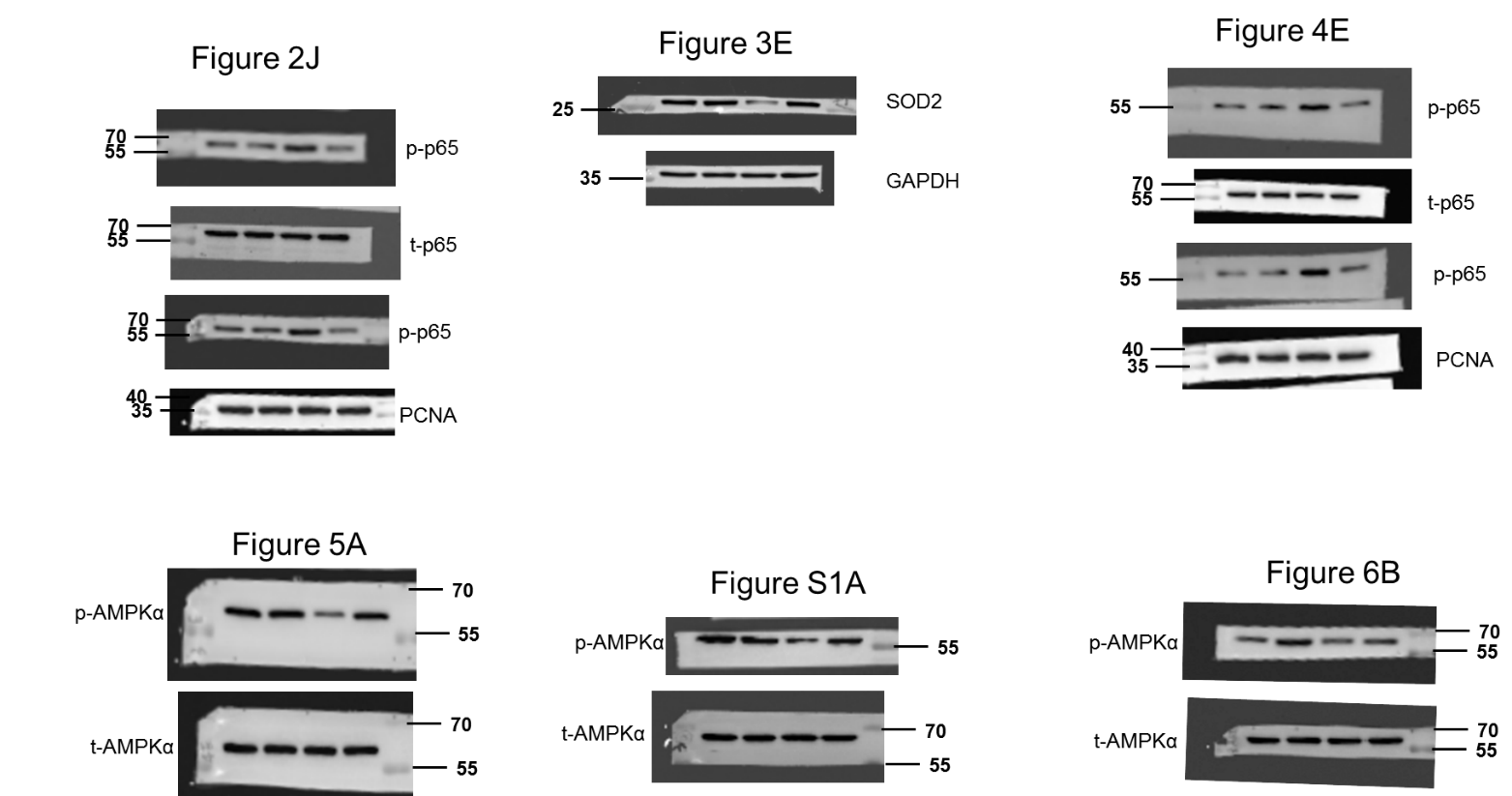
The original western blot images related to different figures.


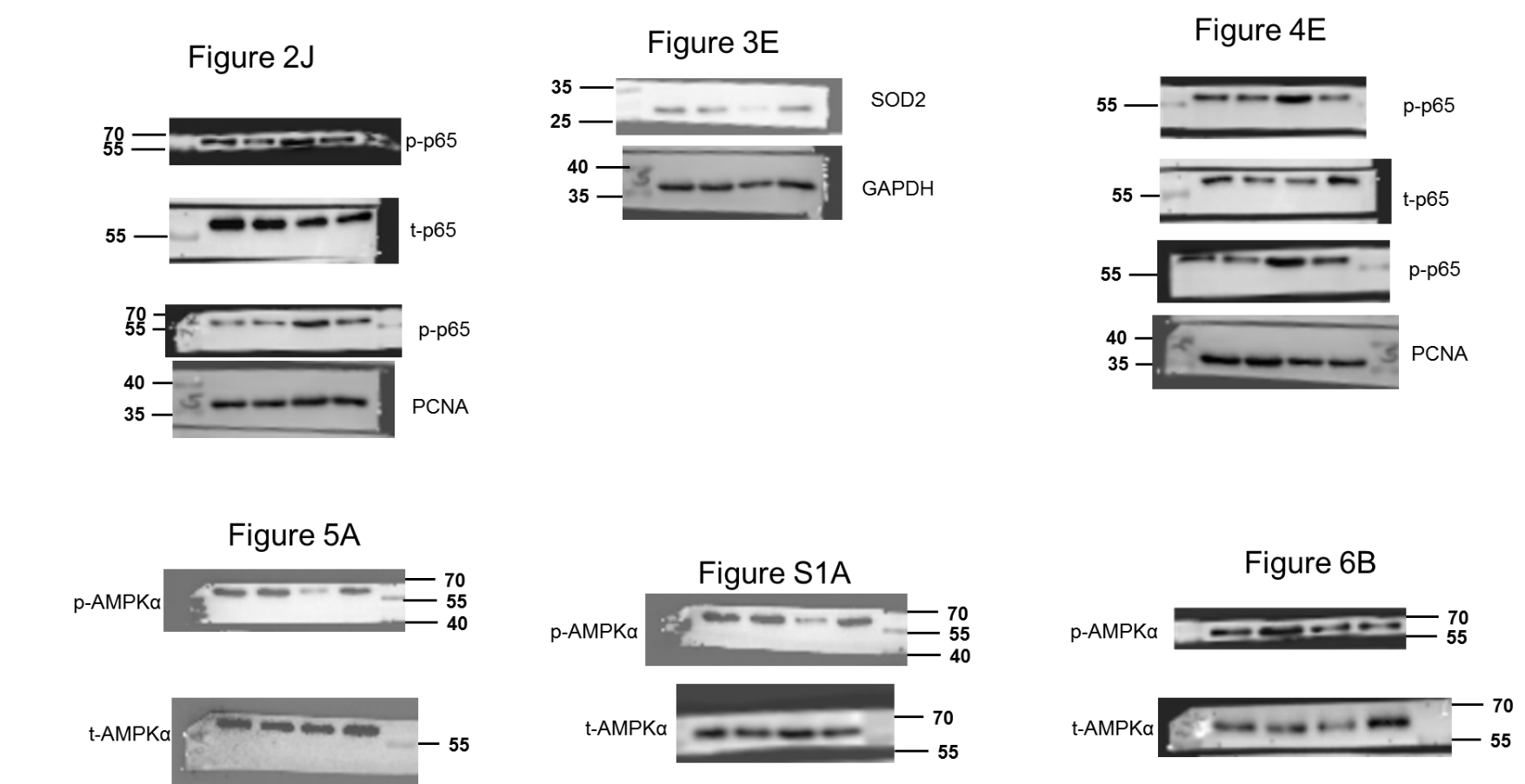


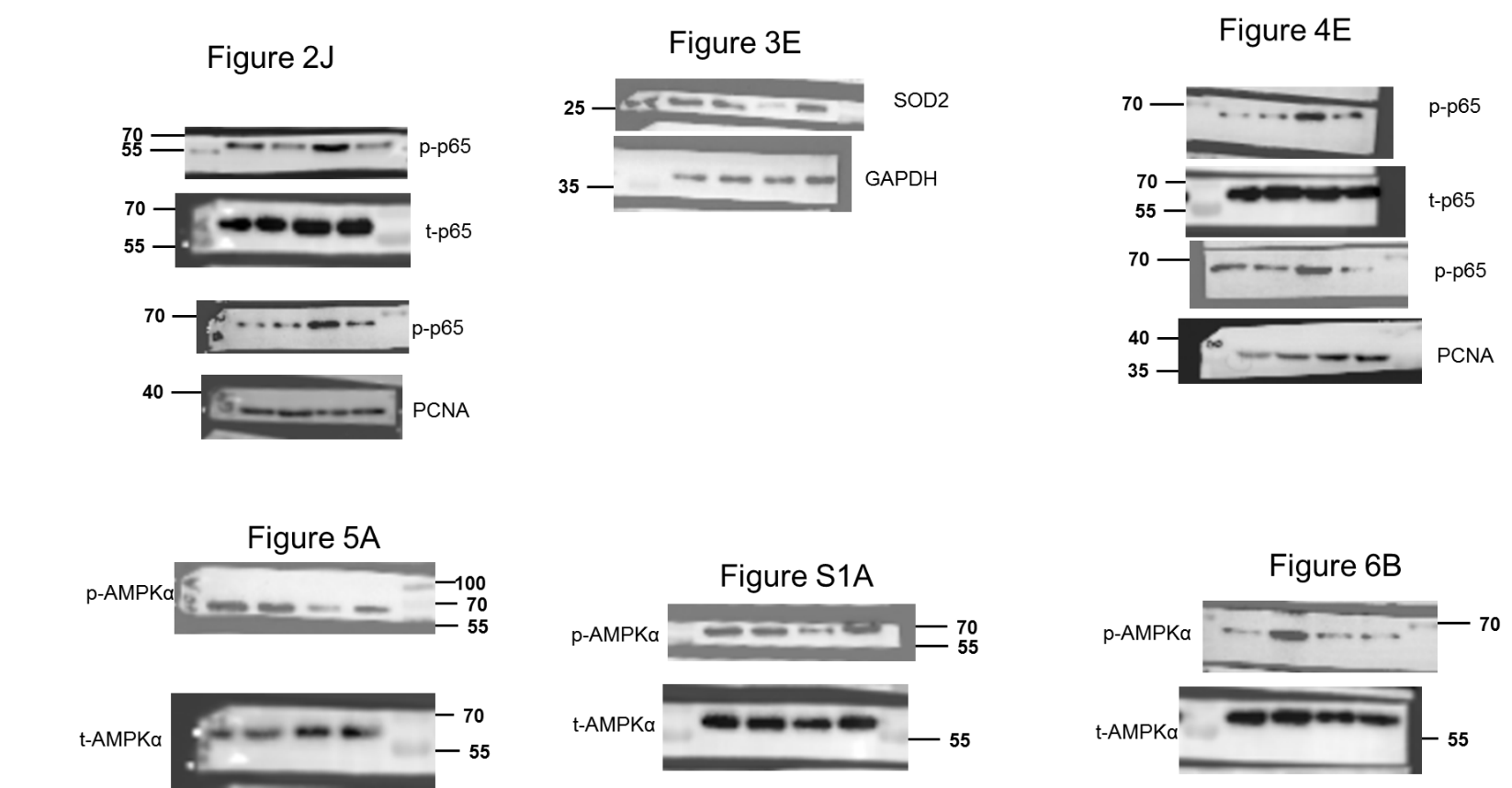

Supplement: Supplementary file 1 — Supplementary Material 1 [file 40360_2023_698_MOESM1_ESM.docx]
